# Supplementary material for: Territories of Nerve Endings of the Medial Plantar Nerve within the Abductor Hallucis Muscle: Clinical Implications for Potential Pain Management
Source: Diagnostics (Basel). 2024 Aug 7;14(16):1716. doi: 10.3390/diagnostics14161716 (PMC11354053; doi:10.3390/diagnostics14161716)
Supplement: Supplementary file 1 [file diagnostics-14-01716-s001.zip › diagnostics-3042945-supplementary.pdf]

Supplement Table S1. Cadaver information

	Cadaver No.	Sex	Age
Dissection & Sihler's staining	1	Male	82
	2	Female	86
	3	Male	74
	4	Male	89
	5	Female	64
	6	Male	78
	7	Male	79
	8	Female	87
	9	Male	83
	10	Male	82
Verification of the injection	11	Female	85
	12	Male	74
	13	Female	76
	14	Male	82
	15	Male	65
	16	Female	86
	17	Male	89
	18	Female	73
	19	Male	67
	20	Male	49
